# Understanding and overcoming hybrid lethality in seed and seedling stages as barriers to hybridization and gene flow

**DOI:** 10.3389/fpls.2023.1219417

**Published:** 2023-07-05

**Authors:** Hai He, Kumpei Shiragaki, Takahiro Tezuka

**Affiliations:** ^1^ School of Agriculture, Sun Yat-sen University, Shenzhen, China; ^2^ Laboratory of Plant Breeding and Genetics, Graduate School of Agricultural and Life Sciences, University of Tokyo, Tokyo, Japan; ^3^ Laboratory of Breeding and Genetics, Graduate School of Agriculture, Osaka Metropolitan University, Sakai, Osaka, Japan; ^4^ Education and Research Field, School of Agriculture, Osaka Metropolitan University, Sakai, Osaka, Japan

**Keywords:** Bateson-Dobzhansky-Muller model, endosperm balance number, hybrid seed lethality, hybrid seedling lethality, reproductive isolation

## Abstract

Hybrid lethality is a type of reproductive isolation barrier observed in two developmental stages, hybrid embryos (hybrid seeds) and hybrid seedlings. Hybrid lethality has been reported in many plant species and limits distant hybridization breeding including interspecific and intergeneric hybridization, which increases genetic diversity and contributes to produce new germplasm for agricultural purposes. Recent studies have provided molecular and genetic evidence suggesting that underlying causes of hybrid lethality involve epistatic interaction of one or more loci, as hypothesized by the Bateson–Dobzhansky–Muller model, and effective ploidy or endosperm balance number. In this review, we focus on the similarities and differences between hybrid seed lethality and hybrid seedling lethality, as well as methods of recovering seed/seedling activity to circumvent hybrid lethality. Current knowledge summarized in our article will provides new insights into the mechanisms of hybrid lethality and effective methods for circumventing hybrid lethality.

## 1 Introduction

Distant or wide hybridization including interspecific and intergeneric hybridization increases genetic diversity and contributes to produce new germplasm by transferring resistance to disease or novel and useful phenotypes for agricultural purposes. However, the formation and evolution of reproductive isolation has prevented gene flow between species through distant hybridization (Lafon-Placette et al., 2017; Kulmuni et al., 2020). Despite its evolutionary importance, reproductive isolation is also an obstacle to distant hybridization breeding in plants. Reproductive isolation involves various pre-mating, post-mating prezygotic, and postzygotic isolating barriers in plants (Coyne and Orr, 2004; Savolainen et al., 2006; Rieseberg and Willis, 2007; Rieseberg and Blackman, 2010). Ecogeographic isolation and pollinator isolation (pollinator fidelity in a natural mixed population) are typical examples of pre-mating isolation barriers (Ramsey et al., 2003; Coyne and Orr, 2004; Savolainen et al., 2006; Streisfeld and Kohn, 2007; Zhang et al., 2022a), whereas interspecific pollen-pistil incompatibility, conspecific pollen precedence, gametic incompatibility, and pistil-length mismatch are examples of post-mating prezygotic isolating barriers (Rieseberg and Willis, 2007; Lee et al., 2008; Rieseberg and Blackman, 2010; Huang et al., 2023). Postzygotic isolation barriers include hybrid seed lethality (Bikard et al., 2009; Dziasek et al., 2021), immature fruit abscission (Gupta et al., 1996; He et al., 2019; Kawaguchi et al., 2021), hybrid seedling lethality or inviability (Tezuka et al., 2010; Tezuka, 2013; Xiao et al., 2017; Deng et al., 2019; Mino et al., 2022), hybrid weakness (Ichitani et al., 2011; Shiragaki et al., 2020b), hybrid sterility (Yamagata et al., 2010; Koide et al., 2018; Li et al., 2020), and hybrid breakdown (Li et al., 1997; Kubo and Yoshimura, 2005; Zhang et al., 2021).

Hybrid lethality has been widely studied in various plant species, including genera *Nicotiana* (Tezuka et al., 2010; He et al., 2019), *Arabidopsis* (Bomblies et al., 2007; Dilkes et al., 2008), *Oryza* (Nadir et al., 2018), *Triticum* (Hermsen, 1963b; Tikhenko et al., 2005), and *Capsella* (Dziasek et al., 2021). Hybrid lethality can be observed in hybrid embryos during seed development and in hybrid seedlings or plants during plant development without infection. Hybrid lethality in embryo results in seed abortion and is often called hybrid seed lethality. Regarding hybrid lethality in seedlings, different terms, such as hybrid weakness, hybrid necrosis, or others, are used depending on the severity of the symptom and plant species. Because of the different growth and developmental stages during which hybrid lethality is observed, the similarities and differences between hybrid seed lethality and hybrid seedling lethality are topics of interest for future research.

**Figure 1:**
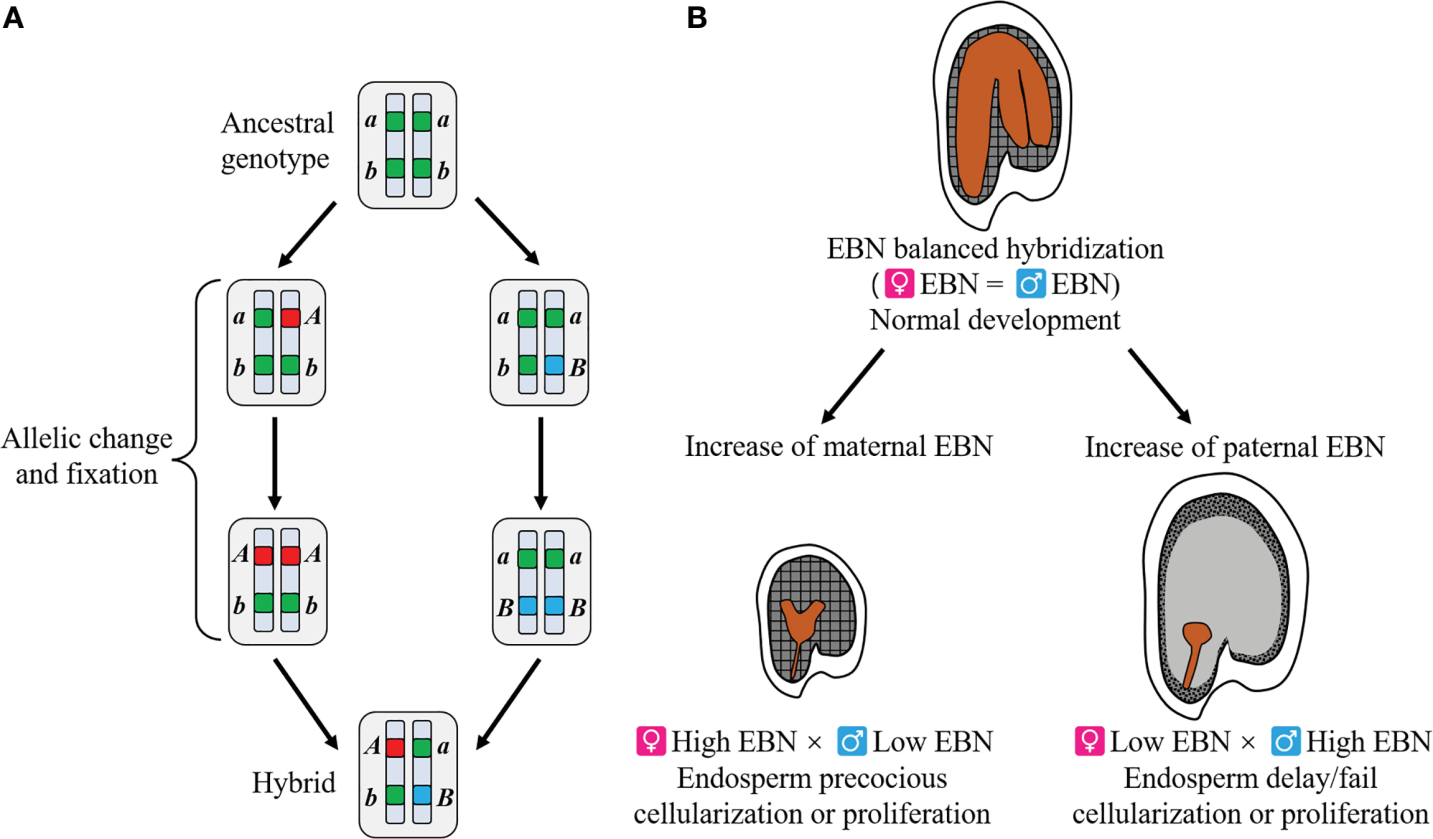
Overview of two theories explaining genetic incompatibilities. **(A)** Bateson–Dobzhansky–Muller model. An ancestral population showed the genotype of *aabb*. Each lineage evolved to independent mutations (*a* to *A* and *b* to *B*). When hybridization occurs between the newly formed two species, deleterious interaction of the two alleles, *A* and *B*, causes genetic incompatibilities. In this model, allelic interaction of a single locus as well as two or more loci might be responsible for genetic incompatibilities. **(B)** Endosperm balance number (EBN) hypothesis. Balanced effective ploidy or EBN between parents yields normal endosperm, leading to normal embryo development and thus normally germinable seeds. When EBN of parents is unbalanced, the timing of endosperm cellularization or proliferation is disturbed, resulting in embryo arrest and hybrid seed lethality. This hypothesis is applicable to both interspecific and interploidy hybridizations.

Recent evidence has shown that hybrid lethality is often caused by epistatic gene interaction of two or more loci, as explained by the Bateson–Dobzhansky–Muller (BDM) model (**Figure 1A**) (Johnson and Porter, 2000; Johnson, 2002; Bomblies and Weigel, 2007; Sweigart and Willis, 2012). In this model, hybrid lethality is caused by genes that were present in an ancestral population but evolved differences in isolated descendent populations. Hybrid lethality in seeds and seedlings may be caused by developmental defects due to genes encoding disease resistance proteins (Bomblies et al., 2007; Chen et al., 2014; Deng et al., 2019), such as duplicate copies of critical genes for development, which were formed during divergent evolution, including photosynthetic gene (Zuellig and Sweigart, 2018) and histidinol-phosphate amino-transferase gene (*HPA*) (Bikard et al., 2009).

In this review, we focused on and summarized the current knowledge on hybrid lethality in seeds and seedlings, particularly but not exclusively in *Nicotiana* (tobacco) species. We also introduce recent molecular advances for finding similarities and/or differences in mechanisms between both types of hybrid lethality. Finally, we discuss the methods to overcome or circumvent hybrid lethality and the active application of hybrid lethality to prevent gene flow.

## 2 Hybrid seed lethality

### 2.1 Two categories of hybrid seed lethality

Hybrid seed lethality during seed development is observed after successful fertilization in both interspecies and interploidy hybridizations and has been well studied in *Nicotiana* (Kostoff, 1930; McCray, 1933; He et al., 2020), *Arabidopsis* (Bikard et al., 2009; Lafon-Placette et al., 2018), *Oryza* (Ishikawa et al., 2011), *Triticum* (Tikhenko et al., 2008; Tikhenko et al., 2017a), and *Mimulus* (Coughlan et al., 2020). Hybrid seed lethality is characterized by embryo arrest and/or endosperm defects. Based on underlying causes, hybrid seed lethality is classified into two main categories: (1) hybrid seed lethality with underlying causes in embryo itself (impaired or arrested embryo development, but normal endosperm development are observed), and (2) hybrid seed lethality caused by defective endosperm development, leading to embryo arrest (**Table 1**).

**Table 1:** Summary of hybrid seed lethality described in this review.

Genus	Phenotype	Models	Methods to overcome or circumvent hybrid lethality						References
			(A)	(B)	(C)	(D)	(E)	(F)	
*Arabidopsis*	Embryo arrest	BDM	ND	ND	ND	ND	ND	ND	Bikard et al. (2009)
*Triticum*	Embryo arrest	BDM	ND	Ineffective	ND	ND	ND	ND	Tikhenko et al. (2005); Tikhenko et al. (2008); Tikhenko et al. (2010); Tikhenko et al. (2011)
*Arabidopsis*	Endosperm failure	EBN	ND	ND	Effective	Effective	Effective	Effective	Scott et al. (1998); Bushell et al. (2003); Josefsson et al. (2006); Lafon-Placette et al. (2017); Huc et al. (2022); Xu et al. (2023)
*Capsella*	Endosperm failure	EBN	ND	ND	Effective	ND	Effective	ND	Rebernig et al. (2015); Lafon-Placette et al. (2018); Huc et al. (2022)
*Solanum*	Endosperm failure	EBN	ND	ND	ND	ND	ND	ND	Roth et al. (2018)
*Mimulus*	Endosperm failure	EBN	ND	ND	ND	ND	ND	ND	Oneal et al. (2016); Garner et al. (2016); Coughlan et al. (2020)
*Nicotiana*	Endosperm failure	EBN	ND	Effective or ineffective (depending on types of hybrid seed lethality)	Effective	ND	ND	ND	Iwai et al. (1986); Subhashini et al. (1986); He et al. (2019); He et al. (2020)
*Oryza*	Endosperm failure	EBN	ND	ND	Effective	Effective	ND	ND	Köhler et al. (2010); Ishikawa et al. (2011); Schatlowski and Köhler (2012); Sekine et al. (2013); Tonosaki et al. (2018)

ND, no data; BDM, Bateson–Dobzhansky–Muller; EBN, endosperm balance number.

(A), Irradiation; (B), Tissue culture; (C), Ploidy manipulation; (D), Modification of gene expression; (E), Application of methyltransferase inhibitor; (F), Abscisic acid.

#### 2.1.1 Hybrid seed lethality with underlying causes in the embryo itself

Several studies have reported that early embryo abnormal development is the main cause of hybrid seed lethality in *Arabidopsis* (Bikard et al., 2009) and tribe *Triticeae* (Tikhenko et al., 2008). In intergeneric hybridization between wheat (*Triticum durum*) and rye (*Secale cereale*), both belonging to tribe *Triticeae*, despite normal endosperm development, the development of shoot apical meristem (SAM) in hybrid embryo is arrested, leading to incompletely differentiated embryos and non-germinating seeds (Tikhenko et al., 2005; Tikhenko et al., 2008). Hybrid embryo lethality in this cross is caused by the interaction between two alleles from two loci, i.e., wheat *embryo lethality* (*Eml*)*-A1* and rye *Eml-R1*, which are likely to be orthologous genes (Tikhenko et al., 2010; Tikhenko et al., 2011). Gene mapping results implied that *Eml-A1* is involved in SAM maintenance, and thus, the underlying cause of hybrid seed lethality is the embryo itself (Tikhenko et al., 2017a).

Similarly, in hybridization between *Arabidopsis thaliana* accessions Columbia (Col) and Cape Verde Islands (Cvi), embryo arrest, resulting in hybrid seed lethality, is caused by duplicate genes LD1.1 and LD 1.5, which encode histidinol-phosphate amino-transferase that catalyzes an important step in the biosynthetic pathway leading to an essential amino acid histidine (Bikard et al., 2009). Homozygous states for both silenced Col allele at the LD1.1 locus and silenced Cvi allele at the LD1.5 locus causes embryo lethality (Bikard et al., 2009). Hence, hybrid seed lethality with underlying causes in embryo agrees with the BDM model and, at least in some cases known so far, is caused by gene functional alteration.

#### 2.1.2 Hybrid seed lethality with underlying causes in endosperm

Polyploidy is widely acknowledged in plant evolution. Although several mechanisms are considered to be related to polyploidization, the most likely mechanism is the participation of unreduced 2*n* gametes. However, production of polyploids through combination of *n* and *2n* gametes is often prevented. Even if a few polyploids survive, the polyploids have difficulty producing viable offspring in hybridization with diploid progenitors. This reproductive barrier, which is called the triploid block, often involves incompatibility in the endosperm or hybrid seed lethality and is considered to act as an instant reproductive barrier (Köhler et al., 2010; Schatlowski and Köhler, 2012).

Research to date has implicated that in many cases, hybrid seed lethality is caused by abnormal endosperm development (Scott et al., 1998; Bushell et al., 2003; Ishikawa et al., 2011; Rebernig et al., 2015; Lafon-Placette et al., 2017). In angiosperms, endosperm is essentially a triploid tissue developed after the fusion of two polar nuclei with a sperm nucleus and is mainly responsible for providing nutrition for embryo growth and germination. Based on differences in its developmental patterns, endosperm is classified into three types: free nuclear, *ab initio* cellular, and helobial (Vijayaraghavan and Prabhakar, 1984; Floyd and Friedman, 2000). Hybrid seed lethality has been studied in plants with free nuclear or *ab initio* cellular endosperm. In free nuclear endosperm, fertilized triploid endosperm nucleus divides without formation of cell wall and later become cellularized, whereas the cell wall is coordinately formed with every endosperm nucleus division in *ab initio* cellular endosperm. In plants with free nuclear endosperm, hybrid seed lethality is characterized by a disturbance in the timing of endosperm cellularization, which is an important developmental transition stage for embryo development in this type of endosperm (Ishikawa et al., 2011; Sekine et al., 2013; Lafon-Placette et al., 2017; İltaş et al., 2021). In the *ab initio* cellular endosperm, hybrid seed lethality shows impaired endosperm cell proliferation (Oneal et al., 2016; Roth et al., 2018).

##### 2.1.2.1 Endosperm balance number is involved in hybrid seed lethality

Hybrid seed lethality caused by defective endosperm development is well explained by effective ploidy or endosperm balance number (EBN) hypothesis proposed by Johnston et al. (1980) (**Figure 1B**). EBN is an arbitrary number allocated to each species and the normal development of endosperm requires a relative maternal (m):paternal (p) EBN ratio of 2:1 (Johnston et al., 1980; Städler et al., 2021). In intraspecies-interploidy hybridization, m:p genome ratio deviates from 2:1; thus, EBN ratio also deviates from 2:1, resulting in endosperm developmental failure. Maternal EBN excess generally results in precocious developmental transition in the endosperm and the production of smaller seeds compared to self-pollinated parental seeds, whereas paternal EBN excess generally results in delayed or failed endosperm development and the production of bigger seeds. Such examples that can be explained by the EBN hypothesis have been reported in many plant species including *Arabidopsis* (Scott et al., 1998), *Oryza* (Sekine et al., 2013), and *Mimulus* (Coughlan et al., 2020). Similarly, the EBN hypothesis fits well with hybrid seed lethality in interspecies hybridizations, even if the parental species have the same ploidy level (Scott et al., 1998; Rebernig et al., 2015; Garner et al., 2016). Therefore, differences in parental EBN rather than differences in parental ploidy levels are assumed to be responsible for hybrid seed lethality.

*Nicotiana* species have *ab initio* cellular endosperm (Sehgal and Gifford, 1979). In this genus, hybrid seed lethality is widely observed in interspecies hybridizations (McCray, 1933; Reed and Collins, 1978; Subhashini et al., 1985; Iwai et al., 1986; Subhashini et al., 1986; He et al., 2020). Recently, two types of hybrid seed lethality, types I and II hybrid seed lethality, which show different severity of symptoms dependent on the maternal accessions, were reported in hybridizations between octoploid *Nicotiana suaveolens* and allotetraploid *N. tabacum* (He et al., 2020). Type I seed lethality occurs in hybridization between *N. suaveolens* PI 555561 (8x) and *N. tabacum* (4x) and is characterized by precocious developmental transition and subsequent developmental arrest of the endosperm and abnormal hypertrophy of the embryo remaining in globular state. In contrast, type II seed lethality occurs in another hybridization between *N. suaveolens* accession PI 555565 (8x) and *N. tabacum* (4x) and is characterized by symptoms more severe than those of type I seed lethality, such as precocious developmental transition of the endosperm and subsequent narrowing of the endosperm region as if pressed by surrounding cells, and embryo growth arrest in the early globular stage. Both type I and type II seed lethality are related to maternal ploidy level as witnessed by the experiments using ploidy manipulated lines. Even in the case of hybridization producing normal seeds, phased increase of ploidy levels in maternal plants subsequently causes type I and type II seed lethality in both interspecies and interploidy hybridizations. The two types of hybrid seed lethality are reversed by an increase in ploidy levels of paternal parents. Thus, hybrid seed lethality in *Nicotiana* is well explained by EBN hypothesis and higher ploidy levels in maternal parents than in paternal parents cause type I or type II seed lethality (He et al., 2020; He et al., 2022).

Previous studies have demonstrated that parent-of-origin specific hybrid endosperm defect is observed in hybrid seed lethality in reciprocal hybridizations and have implied that genomic imprinting is involved in the endosperm defect (Bushell et al., 2003; Josefsson et al., 2006; Lafon-Placette et al., 2017). Genomic imprinting is the epigenetic phenomenon modifying the expression of genes in a parent-of-origin manner. Genomic imprinting mainly includes DNA methylation, histone modification, and non-coding RNA regulation, and disturbed balance of these modification is expected to cause hybrid seed lethality (Lauria et al., 2004; Erdmann et al., 2017; Jiang et al., 2017; Martinez et al., 2018; Dziasek et al., 2021). Current epigenetic analysis in plants suggested that the regulation of imprinting in plants is likely to be explained through a combination of several different epigenetic mechanisms, and these mainly include DNA methylation and trimethylation of lysine 27 of histone H3 (H3K27me3) (reviewed by Batista and Köhler (2020)).

EBN might be related to parental conflict hypothesis, where maternal and paternal genomes have opposite effects on offspring development: the maternal parent is equally related to all of their progeny and thus should allocate equally, whereas the paternal parents are only related to their own progeny, but not to the competing half-siblings, and thus should somehow direct the maternal parent to allocate differentially (Haig and Westoby, 1989; Haig and Westoby, 1991). In plants, the parental conflict hypothesis predicts that the maternal genome excess or maternally expressed genes (MEGs) lead to reduced nutrient flow to the embryo, reduced seed size, and potentially reduced seed set under unfavorable conditions. Conversely, the paternal genome excess or paternally expressed genes (PEGs) promotes nutrient flow to the embryo and increases seed size and seed set (Schatlowski and Köhler, 2012; Bai and Settles, 2015). This is consistent with the endosperm and embryo observation in interploidy hybridizations using parental plants with stepwise changes in EBN (Scott et al., 1998; He et al., 2022).

Several key genes for EBN-based hybrid seed lethality have been identified, suggesting that BDM-type mechanism might be related to endosperm of incompatible species (Köhler et al., 2010). Hybrid seed lethality in paternal excess hybridization can be circumvented by using PEG mutants, such as *PICKLE RELATED 2* (*PKR2* which encodes a CHD3 chromatin remodeler), *PHERES1* (*PHE1*, the type I MADS-box transcription factor), and *ADMETOS* (*ADM* which encodes a protein belonging to the diverse family of molecular chaperones called J-domain proteins) (Kradolfer et al., 2013b; Huang et al., 2017; Batista et al., 2019). In contrast, MEG mutants, such as *MEDEA* (*MEA*, the Polycomb group gene), are effective for circumventing hybrid seed lethality in maternal excess hybridization (Kradolfer et al., 2013a). Additionally, the mutant for *TRANSPARENT TESTA GLABRA2* (*TTG2;* which encodes a WRKY transcription factor controlling epidermal cell fate with pleiotropic effects on seed development and trichome production) has also been reported to partially rescue hybrid seeds from lethality through reduced integument cell elongation and precocious endosperm cellularization in paternal excess hybridization (Garcia et al., 2005; Dilkes et al., 2008). Similarly, the mutant for *TRANSPARENT TESTA 4* (*TT4;* which encodes the enzyme chalcone synthase) has also been reported to rescue hybrid seeds in paternal excess hybridization (Scott et al., 2013; Doughty et al., 2014). The *ttg2* and *tt4* mutants are characterized by the lack of flavonoid seed pigmentation in the seed coat because they do not accumulate proanthocyanidins, suggesting the involvement of flavonoid biosynthesis pathway in hybrid seed lethality (Scott et al., 2013). A recent study demonstrated that mutants of other genes in the flavonoid biosynthesis pathway also rescued hybrid seeds from lethality; in particular, *tt8* mutants completely rescued hybrid seeds unlike mutants of other genes, suggesting that auxin flux and signaling, rather than flavonoid biosynthesis pathway, might be involved in hybrid seed lethality because *TT8* interacts with genes expressed in seed coat including the repressor IAA27/PAP2, a canonical Aux/IAA (Zumajo-Cardona et al., 2023). However, despite these extensive studies, underlying molecular mechanisms of EBN differences are largely unknown. Hence, elucidating the detailed mechanism of hybrid seed lethality caused by endosperm defect in interspecific and interploidy hybridizations remains challenging.

##### 2.1.2.2 Immature fruit abscissions caused by hybrid seed lethality

Plants have evolved sophisticated organ abscission to respond to seed/fruit dispersal, pathogen attack, and environment stress. Abscission is a universal and physiological process in plant development that occurs through loosening of adjacent cell walls within the abscission zone (AZ) and subsequent cell separation (Addicott, 1982; Taylor and Whitelaw, 2001). It takes place under various developmental signals, such as fertilization, senescence, and ripening, and environmental signals, such as light, pathogen, and temperature (reviewed by Sawicki et al. (2015)). Abscission is also observed in developing ovaries (immature fruits) in interspecies and interploidy hybridizations in *Nicotiana*, which exhibit type II hybrid seed lethality (He et al., 2020; He et al., 2022). The post-pollination developing fruit or pod abscission has also been reported in other interspecific crosses of the genera *Cicer* (Mallikarjuna, 1999), *Lupinus* (Gupta et al., 1996), *Phaseolus* (Mok et al., 1978), and *Vigna* (Barone et al., 1992), all of which belong to the Fabaceae family. Studies in *Nicotiana* have clearly demonstrated that manipulation of parental ploidy levels not only affects hybrid seed lethality (type II, not type I, in the *Nicotiana* case) but also affects immature fruit abscission in interspecies and interploidy hybridizations (He et al., 2020; He et al., 2022). This implies that the parental conflict hypothesis could also be applied to fruit maturation and abscission, which are maternal processes. However, the detailed process by which hybrid seed lethality causes immature fruit abscission is unclear.

Failure of embryo development (seed abortion) may cause ovary or fruit abscission during plant reproduction (Sawicki et al., 2015). In general, plant hormones, such as auxin, ethylene, gibberellins (GAs), and abscisic acid (ABA), play important roles in organ abscission. Ethylene and ABA act as abscission-accelerating hormones (Vernieri et al., 1992; Zacarias et al., 1995; Taylor and Whitelaw, 2001; Zhu et al., 2010), whereas auxin and GAs are well-known inhibitors of abscission (Mahouachi et al., 2009; Basu et al., 2013; Liang et al., 2020). In particular, the interaction between auxin and ethylene plays important roles in regulation of abscission (Taylor and Whitelaw, 2001). A basipetal auxin flux through AZ is considered to prevent abscission by rendering the AZ insensitive to ethylene. When seed abortion occurs, the auxin flux is suppressed, leading to the enhancement of the AZ sensitivity to ethylene and activation of AZ (Sawicki et al., 2015). Similarly, it has been demonstrated that auxin is a trigger of seed development in many plant species; auxin biosynthesis genes are activated after fertilization, then auxin is accumulated and auxin signaling is activated in seed tissues (reviewed by Figueiredo and Kohler (2018)). Fruit abscission occurs when only small number of seeds are contained in the fruit owing to the insufficient supply of auxin from seeds or ovary (Din et al., 2019). Therefore, immature fruit abscission in interspecies and interploidy hybridizations may also be controlled by auxin and ethylene. In the case of *Nicotiana*, when the ovary was occupied by seeds exhibiting type I hybrid seed lethality, a sufficient amount of auxin to prevent immature fruit abscission might be somehow transported to the AZ. On the other hand, when the ovary was occupied by seeds exhibiting type II hybrid seed lethality, the supply of auxin from seeds or ovary might be insufficient, leading to immature fruit abscission. Although involvement of auxin in immature fruit abscission has been suggested because immature fruit abscission, but not type II hybrid seed lethality, was suppressed by exogenous auxin treatments (He et al., 2022), further studies are needed to validate this hypothesis.

### 2.2 Methods of recovering seed activity to circumvent hybrid seed lethality

**Figure 2:**
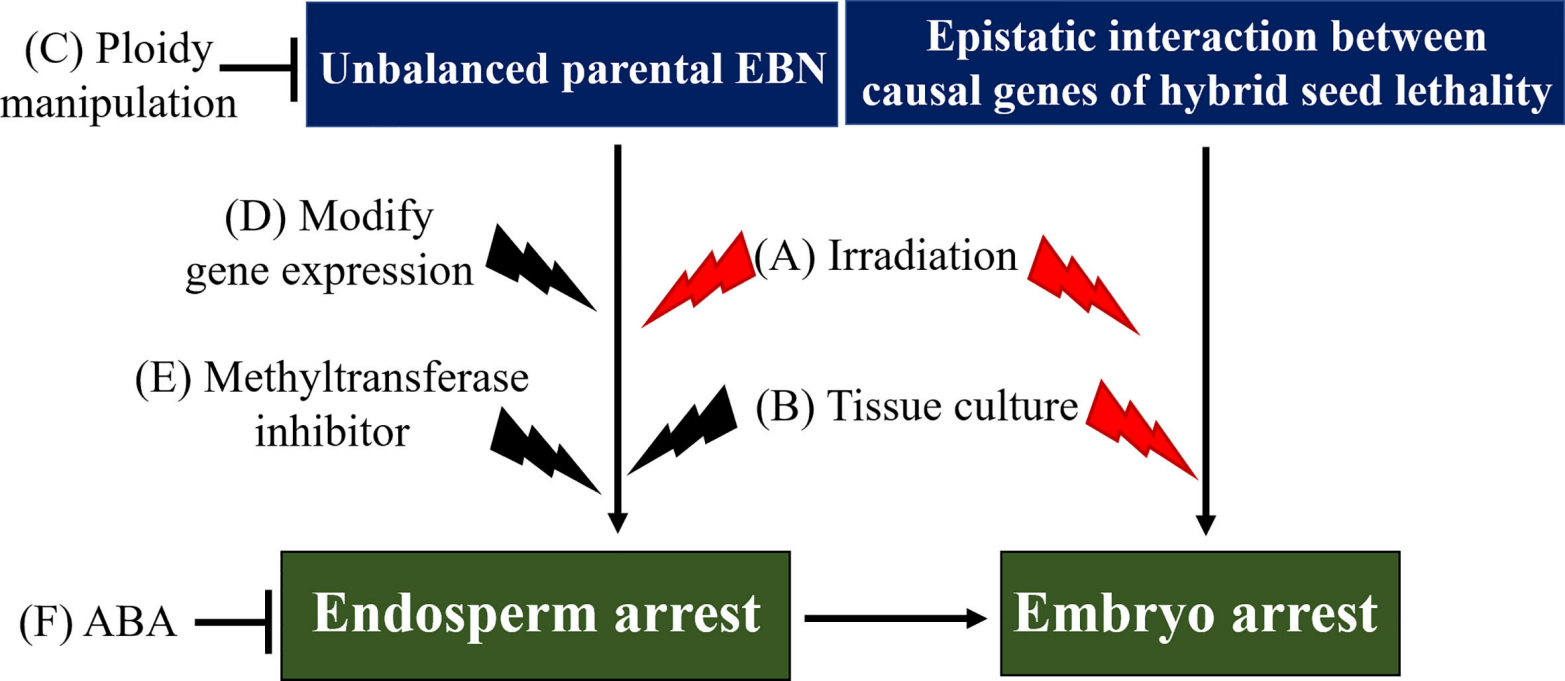
Model of hybrid seed lethality and methods to overcome or circumvent hybrid seed lethality. Two pathways are involved in the induction of hybrid seed lethality: (1) epistatic interaction between causal genes of hybrid seed lethality directly affecting hybrid embryos, and (2) unbalanced parental EBN, which causes endosperm arrest, leading to hybrid embryo arrest. Irradiation **(A)** and tissue culture (embryo rescue technique, **(B)** may be effective to overcome hybrid seed lethality caused by epistatic interaction between causal genes. Tissue culture and ploidy manipulation **(C)** are widely used to overcome or circumvent EBN-based hybrid seed lethality. Additionally, modification of gene expression **(D)**, increase in endogenous ABA levels **(F)**, application of methyltransferase inhibitor **(E)** and exogenous ABA, and possibly irradiation may be effective in some hybridization combinations at least. Black thunderbolt icons represent induction of mutations. Possible methods to overcome hybrid seed lethality are represented by red thunderbolt icons. EBN, endosperm balance number; ABA, abscisic acid.

Circumventing hybrid seed lethality is valuable in achieving distant hybridization to transfer desirable genes to crop germplasm. For example, genes of rye (*S. cereale*) have been introduced into wheat (*T. durum*) germlines to improve resistance to pathogens and pests (reviewed by Crespo-Herrera et al. (2017)). Hence, to circumvent hybrid seed lethality, several methods have been developed, including (1) hybrid embryo rescue technique, and (2) restoration of endosperm development to indirectly rescue the hybrid embryo (**Figure 2**, **Table 1**).

#### 2.2.1 Hybrid seed rescue through the embryo rescue technique

Before hybrid embryos become defective, the immature embryos are isolated and cultured on the medium for their recovery and growth. This embryo rescue technique is effective to rescue embryos from the endosperm defective hybrid seeds and has been widely used in various plant species, including wheat (Polgári et al., 2014) and melon (Nuñez-Palenius et al., 2006). When embryos are too small to isolate, ovule or ovary culture is available for hybrid embryo rescue (**Figure 2B**). For example, in several interspecific hybridizations in *Nicotiana*, ovule culture was successful to obtain hybrid seedlings by circumvent hybrid seed lethality (Reed and Collins, 1978; Shizukuda and Nakajima, 1982; Subhashini et al., 1985; Iwai et al., 1986; Subhashini et al., 1986; Chung et al., 1988). In interspecific hybridization of *Lilium*, embryo rescue by ovary culture was effective to obtain hybrid seedlings (Fernandez et al., 1998). Effectiveness of embryo rescue appears to depend on the severity of hybrid seed lethality because ovule culture was successful in type I hybrid seed lethality but not in type II hybrid seed lethality in *Nicotiana* interspecific-interploidy hybridizations (He et al., 2020). Based on the nutritional requirements of developing embryo, embryonic development is divided into two phases, a heterotrophic phase and autotrophic phase. The embryo is still heterotrophic at the globular stage, and only in the late heart-shaped stage, along with the beginning of cotyledonary development and the consequent internal differentiation, the embryo becomes autotrophic (Raghavan, 1966). Embryo rescue is usually conducted in the heterotrophic phase, where the young embryo still depends at the expense of the endosperm, and can be accomplished *in vitro* by providing, through culture medium, the complex nutritional factors, including amino acids, carbohydrates, vitamins, and other growth factors that would normally be supplied by endosperm. Hence, embryo rescue technique is an effective way to circumvent hybrid seed lethality due to endosperm defective.

In the case where the causal genes are directly involved in embryo development, embryo rescue might not be effective. When embryo rescue was applied to circumvent hybrid seed lethality caused by *Eml-A1* and *Eml-R1* in hybridization between wheat (*T. aestivum*) and rye (*S. cereale*), no positive effect was observed on embryo development (Tikhenko et al., 2008). This further implies that embryo rescue technique is only effective when used to complement the role of the endosperm in supporting the developing embryo.

#### 2.2.2 Hybrid seed rescue through endosperm restoration

Restoration of endosperm development to recover the embryo viability is widely applied in the interploidy and interspecific hybridization. Endosperm developmental defect can be bypassed by altering the EBN of parental species by ploidy manipulation (**Figure 2C**) (Johnston and Hanneman, 1982; Bushell et al., 2003; Lafon-Placette et al., 2017). In interspecific-interploidy hybridization between *N. suaveolens* (8x) and *N. tabacum* (4x) (*ab initio* cellular endosperm), type I and type II seed lethality could be overcome through restoring endosperm and embryo development by increasing paternal *N. tabacum* ploidy from 4x to 8x (He et al., 2020). Another study provides evidence that decreasing maternal EBN can also restore endosperm development and hybrid seed lethality in *Nicotiana* (He et al., 2022).

As described above, endosperm abnormal development responsible for hybrid seed lethality in nuclear endosperm is well studied in *Arabidopsis* and *Oryza*. In this endosperm developmental mode, a disturbance in the timing of endosperm cellularization is the primary cause observed histologically (Ishikawa et al., 2011; Sekine et al., 2013; Lafon-Placette et al., 2017; İltaş et al., 2021). Hence, recovering the endosperm development by manipulation of parental ploidy levels is effective to support nutrition for embryo growth and germination. In interspecific hybridization between diploid species *O. sativa* and *O. longistaminata*, the manipulation to increase the *O. sativa* ploidy levels restored the endosperm cellularization and hybrid seed viability (Ishikawa et al., 2011; Tonosaki et al., 2018). Additionally, several mutants producing unreduced gametes have been identified (Köhler et al., 2010; Schatlowski and Köhler, 2012). Such a mutants might also be effective to circumvent hybrid seed lethality by balancing EBN ratio in endosperm.

In polyploidization, another mechanism for bypassing triploid block may be polyspermy, a fertilization of one egg with two sperm. In *A. thaliana*, triploid block can be circumvented by polyspermy because polyspermy selectively polyploidizes the egg cell while rendering the genome size of endosperm unaffected (Mao et al., 2020).

Besides ploidy manipulation, chemical treatment can also restore endosperm development. Recent study showed that epimutagenesis chemically induced by treatment with DNA methyltransferase inhibitor 5-azacytidine was successful in circumventing hybrid seed lethality in *Arabidopsis* interploidy hybridization and *Capsella* interspecific hybridization (**Figure 2E**) (Huc et al., 2022). In *Arabidopsis* interploidy hybridization, endosperm cellularization could be restored by increasing endogenous ABA levels using mutants for ABA hydroxylase-encoding gene *CYP707A2* or by exogenous application of ABA, leading to suppression of the triploid block and hybrid embryo arrest (**Figure 2F**) (Xu et al., 2023). Hence, endosperm defects can be restored by ploidy alteration or specific reagent treatment, leading to recovery of embryo viability and circumventing hybrid seed lethality.

Additionally, temperature might influence hybrid seed lethality. In interspecies hybridizations in *Arabidopsis*, hybridizations using parental plants growing at low temperatures resulted in suppression of endosperm-based hybrid seed lethality (Bjerkan et al., 2020). This low temperature effect may be attributed to the increased levels of ABA (Xu et al., 2023).

## 3 Hybrid seedling lethality

### 3.1 Mechanisms of hybrid seedling lethality

Hybrid lethality in seedlings has been reported for over 100 years in many plant species, such as *Triticum*-*Aegilops* complex (Sax, 1921), *Nicotiana* (Malloch and Malloch, 1924; Christoff, 1928; Kostoff, 1930), *Crepis* (Hollingshead, 1930), *Gossypium* (Gerstel, 1954), *Oryza* (Oka, 1957), *Solanum* (Day, 1958; Sawant, 1958), *Cucurbita* (Whitaker and Bemis, 1964), *Capsicum* (Hirose et al., 1960; Pickersgill, 1971), *Papaver* (Mcnaughton and Harper, 1960), *Hordeum* (Takahashi et al., 1970), *Phaseolus* (Shii et al., 1980; Gepts and Bliss, 1985), and *Camellia* (Nadamitsu et al., 1986). Several terms representing hybrid lethality or developmental defects of hybrid seedlings are used by researchers. Although these terms are used differently according to plant species, they seem to be mainly used according to the severity of phenotypic symptoms. The general symptoms of hybrid lethality are apparent death of seedlings and are typically observed in interspecific hybridizations in *Nicotiana* (Yamada et al., 1999; Tezuka, 2012; Tezuka, 2013). Hybrid weakness is characterized by the weak growth of seedlings compared to both parents and is typically observed in *Oryza* (Chu and Oka, 1972; Chen et al., 2013; Nadir et al., 2019; Shiragaki et al., 2019), *Capsicum* (Shiragaki et al., 2020b; Shiragaki et al., 2021; Shiragaki et al., 2022), and *Phaseolus* (Koinange and Gepts, 1992; Reiber and Neuman, 1999a). Hybrid necrosis may refer to both types and is mainly used for the *Triticum*-*Aegilops* complex (Tsunewaki and Kihara, 1962; Toxopeus and Hermsen, 1964; Mizuno et al., 2010; Zhang et al., 2022b) and *Arabidopsis* (Bomblies et al., 2007; Świadek et al., 2017). Such hybrid seedling lethality and other similar phenomena are observed in F_1_ hybrid seedlings. When F_1_ hybrids are normal but their F_2_ and later progeny contain individuals showing seedling lethality, this phenomenon is called hybrid breakdown (hybrid breakdown is also used to refer sterility in such a generation) (Fukuoka et al., 1998; Plötner et al., 2017; Matsubara, 2020). The molecular mechanism of hybrid seedling lethality has been unknown for a long time and has only recently become somewhat clear. Recent research has revealed that developmental defects of seedlings, which has referred to using several terms, could also be a phenomenon with a similar mechanism.

#### 3.1.1 Epistatic interaction causing hybrid seedling lethality

Hybrid seedling lethality is generally genetically simple. In many cases, hybrid seedling lethality is caused by deleterious epistatic interaction of two dominant alleles of different loci as proposed by the BDM model (Hollingshead, 1930; Sawant, 1956; Oka, 1957; Tsunewaki, 1960; Hermsen, 1963a; Takahashi et al., 1970; Chu and Oka, 1972; Shii et al., 1980; Lee, 1981; Hu et al., 2016). Several reports have stated that epistatic interaction of alleles at a single locus causes hybrid seedling lethality as hypothesized by the BDM model (Smith et al., 2011; Chae et al., 2014; Todesco et al., 2014). Similarly, seedling lethality in hybrid breakdown are also caused by epistatic interaction of recessive alleles (Oka, 1957; Matsubara, 2020). Additionally, nuclear-cytoplasmic interaction has been reported to cause hybrid seedling lethality (Inai et al., 1993), but no further data are available. Studies to date have isolated several genes and allowed us to understand hybrid seedling lethality from their functions.

#### 3.1.2 Hybrid seedling lethality related to autoimmune response

Hybrid seedling lethality is observed in many interspecific hybridizations in *Nicotiana* (Tezuka, 2012; Tezuka, 2013). In this genus, five types of hybrid lethality, which exhibit different phenotypic abnormalities, have been recognized (Yamada et al., 1999; Tezuka and Marubashi, 2012). Among the lethality types, type II hybrid seedling lethality has been extensively studied using interspecies hybridizations between cultivated tobacco species *N. tabacum* and its wild relatives. The type II hybrid seedling lethality is characterized by browning of hypocotyl and roots in hybrid seedlings during early growth stages, and the hybrid seedlings eventually die. A series of studies have demonstrated that the type II hybrid seedling lethality is accompanied by vacuole-mediated programmed cell death with features of apoptotic cell death and autophagy (Yamada et al., 2000; Tezuka and Marubashi, 2004; Mino et al., 2005; Tezuka and Marubashi, 2006a; Mino et al., 2007b; Ueno et al., 2016) and is related to ethylene (Yamada and Marubashi, 2003), reactive oxygen species (Mino et al., 2002; Mino et al., 2004), and nitrogen oxide (Yamamoto et al., 2017). Taken together, type II hybrid seedling lethality resembles hypersensitive response or cell death, a type of plant defense response with a rapid localized cell death that occurs at the position of pathogen infection (Balint-Kurti, 2019; Salguero-Linares and Coll, 2019). A mitogen-activated protein kinase (MAPK) cascade that functions in plant immunity is reported to be involved in the type II hybrid seedling lethality (Mino et al., 2007a). Furthermore, it has been revealed that several genes related to disease resistance are involved in the type II hybrid seedling lethality (Mino et al., 2002; Masuda et al., 2007; Shiragaki et al., 2020a).

Identification of the causal gene provides further evidence of the involvement of resistance and immune responses in the type II hybrid seedling lethality in *Nicotiana*. *N. tabacum*, an allotetraploid species with S and T genomes, has an allele at the *Hybrid Lethality 1* (*NtHL1*) locus on chromosome H (Ma et al., 2020) of the T genome, or more likely, on chromosome Q of the S genome (based on the analysis using simple sequence repeat (SSR) markers in the *N. tabacum* linkage map) (Marubashi and Onosato, 2002; Tezuka and Marubashi, 2006b; Tezuka et al., 2007; Tezuka et al., 2010; Tezuka et al., 2012), whereas its wild relatives have *Hla1-1* or other alleles at the *Hybrid Lethality A1* (*HLA1*) locus (Iizuka et al., 2012). Epistatic interaction of the two alleles at the two loci causes the type II hybrid seedling lethality. Recently, *NtHL1* was isolated by transposon tagging; this gene codes a coiled-coil nucleotide-binding site-leucine-rich repeat (CC-NBS-LRR) protein, which is probably involved in disease resistance (Ma et al., 2020). Its counterpart, *HLA1*, has been mapped by the linkage analysis (Tezuka et al., 2021). Identification of the *HLA1* will enable a deeper understanding of the interaction between *NtHL1* and *HLA1*.

Another interesting aspect of the involvement of immune response in type II hybrid seedling lethality has been reported by Katsuyama et al. (2021). The molecular chaperone, heat shock protein 90 (HSP90), and two interacting co-chaperons, required for Mla12 resistance (RAR1) and suppressor of G2 allele of skp1 (SGT1), form a complex that interacts with many NBS-LRRs, also known as NLRs (Kadota et al., 2010; Kadota and Shirasu, 2012; Balint-Kurti, 2019). *Agrobacterium*-mediated transient expression of *RAR1* as well as *SGT1* derived from *N. gossei* (a wild relative of *N. tabacum*) induced cell death in *N. tabacum*, whereas the transient expression of those genes derived from *N. tabacum* did not (Katsuyama et al., 2021). Additionally, a specific inhibitor of HSP90, geldanamycin, suppressed cell death in type II hybrid seedling lethality. These findings suggest the involvement of HSP90-SGT1-RAR1 complex in type II hybrid seedling lethality. Because each species has established an appropriate set of chaperons, the chaperons may be incompatible with those from other species (Katsuyama et al., 2021; Mino et al., 2022).

Similar to *Nicotiana*, hybrid seedling lethality in other genera is often associated with autoimmune response (**Table 2**). In hybrid seedling lethality under a two-locus model, at least one causal gene encodes NLR or leucine-rich repeat receptor-like kinase (LRR-RLK) involved in plant immunity (Bomblies et al., 2007; Chen et al., 2014; Sicard et al., 2015; Deng et al., 2019; Si et al., 2021). In hybrid seedling lethality under a one-locus model, the causal gene also encodes NLR or RLK (Smith et al., 2011; Chae et al., 2014). Physiological and molecular studies have also indicated the involvement of plant defense response in hybrid seedling lethality (Mizuno et al., 2010; Shiragaki et al., 2019; Shiragaki et al., 2020b; Shiragaki et al., 2021; Xiao et al., 2021). Therefore, it is apparent that plant defense systems play an important role in the establishment of hybrid seedling lethality systems, which act as reproductive isolation barriers.

**Table 2:** Summary of hybrid seedling lethality described in this review.

Genus	Autoimmune response	Model	Methods to overcome or circumvent hybrid lethality						References
			(A)	(B)	(C)	(D)	(E)	(F)	
*Arabidopsis*	Related	BDM	ND	ND	ND	Effective	ND	ND	Bomblies et al. (2007); Smith et al. (2011); Chae et al. (2014); Todesco et al. (2014)
*Arabidopsis*	Unrelated	BDM	ND	ND	ND	ND	ND	ND	Bikard et al. (2009)
*Brassica*	Related	BDM	ND	ND	ND	ND	ND	ND	Hu et al. (2016)
*Capsicum*	Related	BDM	ND	ND	ND	Effective	ND	ND	Inai et al. (1993); Sawada et al. (2004); Shiragaki et al. (2020b); Shiragaki et al. (2021)
*Chrysanthemum*/ *Leucanthemum*	Unrelated	ND	ND	ND	ND	ND	ND	ND	Li et al. (2023)
*Gossypium*	Related	BDM	ND	ND	ND	Effective	ND	ND	Lee (1981); Deng et al. (2019)
*Mimulus*	Unrelated	BDM	ND	ND	ND	ND	ND	ND	Zuellig and Sweigart (2018)
*Nicotiana*	Related	BDM	Effective	Effective	Effective	Effective	Effective	Effective	Ternovskii et al. (1972); Lloyd (1975); Ternovskii et al. (1976); Iwai et al. (1985); Deverna et al. (1987); Shintaku et al. (1988); Shintaku et al. (1989); Zhou et al. (1991); Inoue et al. (1994); Inoue et al. (1997); Yamada et al. (1999); Mino et al. (2002); Kitamura et al. (2003); Yamada and Marubashi (2003); Tezuka and Marubashi (2004); Kobori et al. (2007); Masuda et al. (2007); Tezuka (2012); Ma et al. (2020); Shiragaki et al. (2020a); Katsuyama et al. (2021)
*Oryza*	Related	BDM	ND	ND	ND	Effective	ND	ND	Oka (1957); Saito et al. (2007); Chen et al. (2014); Shiragaki et al. (2019); Matsubara (2020)
*Oryza*	Unrelated	BDM	ND	ND	ND	ND	ND	ND	Kubo et al. (2022)
*Phaseolus*	Related	BDM	ND	ND	ND	Effective	ND	Effective	Shii et al. (1980); Shii et al. (1981); Reiber and Neuman (1999b); Hannah et al. (2007)
*Triticum*/ *Aegilops*	Related	BDM	Effective	Effective	ND	Effective	ND	ND	Sharma (1969); Dhaliwal et al. (1986); Chen et al. (1989); Mizuno et al. (2010); Zhang et al. (2022b)

ND, no data; BDM, Bateson–Dobzhansky–Muller.

(A), Irradiation; (B), Tissue culture; (C), Defense factor inhibitor; (D), High temperature; (E), Auxin (F), Cytokinin.

#### 3.1.3 Hybrid seedling lethality not resulting from autoimmune response

Although studies on hybrid seedling lethality other than that involving autoimmune response are less advanced, some studies have been reported. As mentioned above, embryo lethality (hybrid breakdown) is caused by duplicate genes LD1.1 and LD 1.5 in hybridization between *Arabidopsis thaliana* accessions (Bikard et al., 2009). Epistatic interaction of these genes also causes weak growth of seedlings in case of the homozygous for Col allele at the LD1.1 locus and heterozygous (Col and Cvi alleles) at the LD1.5 locus. This is due to the reduced histidine quantity in the seedlings because they have only a single functional copy of histidinol-phosphate amino-transferase gene, and the weak phenotype is restored by supplementation of the histidine (Bikard et al., 2009).

In interspecies hybridization in *Mimulus*, F_2_ hybrid seedlings show lethality (hybrid breakdown) owing to a complete lack of chlorophyll production. The lethality is caused by two duplicate genes of *PLASTID TRANSCRIPTIONALLY ACTIVE CHROMOSOME 14* (*pTAC14*), a gene critical for chloroplast development, and F_2_ hybrid seedlings die when they lack a functional copy of *pTAC14* (Zuellig and Sweigart, 2018). In rice intersubspecific hybridizations, weak growth by hybrid breakdown is caused by duplicate recessive genes *hwe1* and *hwe2*. *HWE1* and *HWE2* encode the Esa1-associated factor 6 (EAF6) protein, which is a component of histone acetyltransferase complexes and possibly plays a pivotal role in the transcriptional regulation of essential genes for plant development. *hwe1* and *hwe2* alleles lack the function and cause hybrid breakdown not resulting from autoimmune response (Kubo et al., 2022).

Relationships between plant growth and hybrid seedling lethality has also been reported in another hybridization. *Chrysanthemum morifolium* and *Leucanthemum paludosum* have different flowering seasons. Intergeneric hybrids of the two species flowered at the two seasons by compound expression of *FLOWERING LOCUS T* (*FT*)-like genes derived from both parents, which leads to short vegetative growth. However, the continuous flowering appeared to cause hybrid weakness (Li et al., 2023). Collectively, lack of key genes or a complex combination of genes for plant growth and development could cause hybrid seedling lethality; however, accumulation of more data is necessary to systematize other mechanisms of hybrid seedling lethality than the autoimmune response mechanism.

### 3.2 Methods to overcome or circumvent hybrid seedling lethality

It seems that when hybrid seedling lethality is caused by lack of important elements for plant growth, supplementing the elements, if possible, could be effective to overcome or circumvent hybrid seedling lethality. However, other methods would be needed to overcome or circumvent hybrid seedling lethality related to autoimmune response. Although several methods have been reported to date, the mechanisms underlying these methods have not been fully elucidated. Here, we introduce the methods to overcome or circumvent hybrid seedling lethality and discuss why these methods are effective.

**Figure 3:**
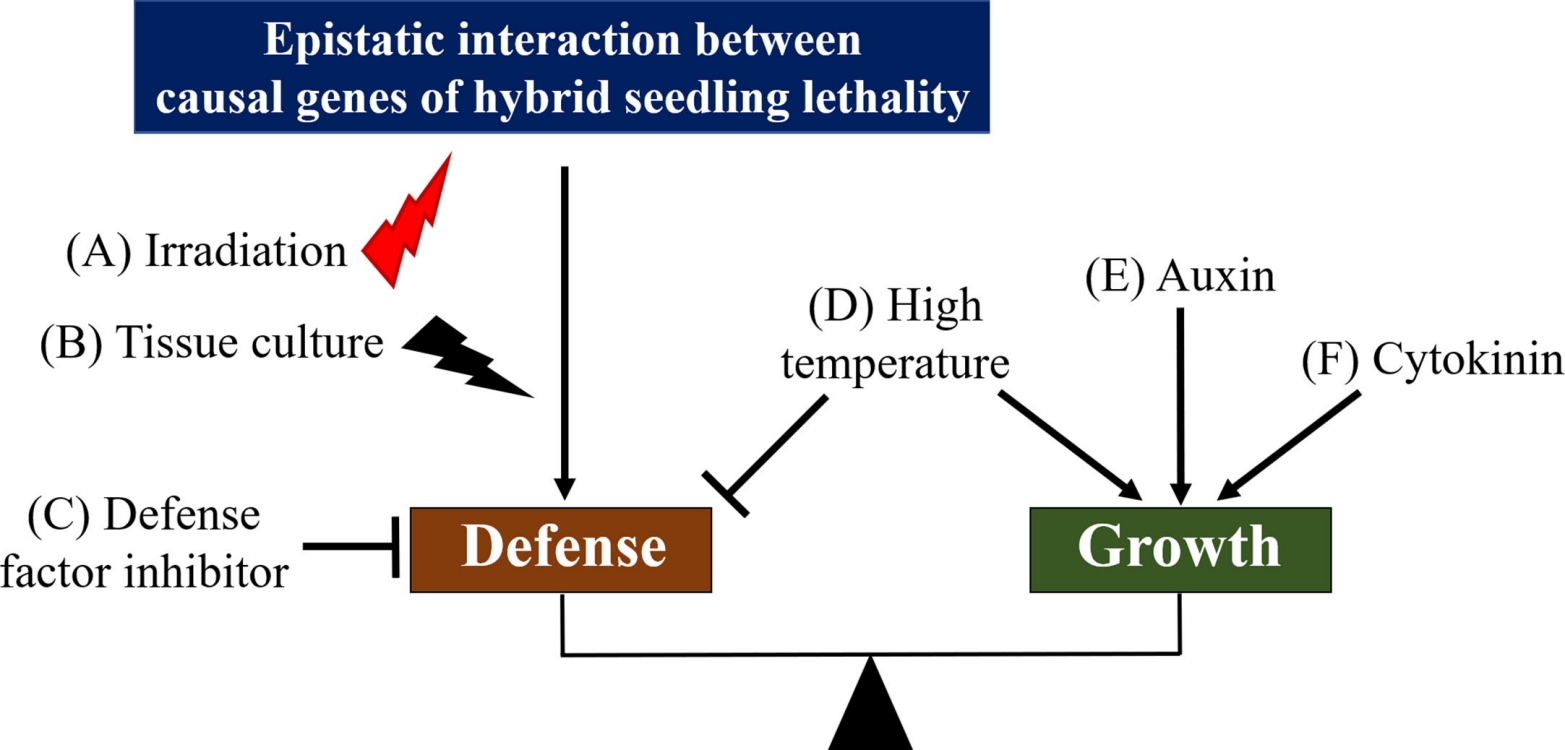
Hypothetical model for the crosstalk between defense and growth pathway in hybrid seedling lethality related to autoimmune response. Known factors or methods to overcome hybrid seedling lethality are shown on the crosstalk diagram. Irradiation **(A)** and tissue culture **(B)** can induce mutations or deletions of causal genes for hybrid seedling lethality or important genes in defense response during hybrid seedling lethality. Application of defense factor inhibitors **(C)**, such as those for phenylalanine ammonia-lyase and ethylene biosynthesis, may be effective to suppress hybrid seedling lethality because these suppress defense response. On the other hand, high temperature **(D)** may suppress defense response and promote seedling growth, and auxin **(E)** and cytokinin **(F)** may activate the growth pathway of seedlings. Effective methods to overcome hybrid seed lethality by irradiation and tissue culture are represented by red (potential induction of mutations) and black (induction of mutations) thunderbolt icons, respectively.

In some methods, hybrid seedling lethality may be suppressed during the crosstalk between defense and growth responses in plants (**Figure 3**, **Table 2**). Elevated temperatures compared with normal growth temperatures suppress hybrid seedling lethality in many cases (Phillips, 1977; Dhaliwal et al., 1986; Tezuka and Marubashi, 2004; Bomblies et al., 2007; Saito et al., 2007; Jeuken et al., 2009; Deng et al., 2019; Shiragaki et al., 2021; Yan et al., 2021); however, in a certain case, low temperature suppressed hybrid seedling lethality in rice (Chen et al., 2014). Several disease resistance responses are temperature-sensitive and suppressed at high temperatures (**Figure 3D**) (Samuel, 1931; Erickson et al., 1999; Sawada et al., 2004). Phytochrome B acting as a temperature sensor is biologically active by high temperature and can activate the expression of PHYTOCHROME INTERACTING FACTOR 4, which promotes growth and suppresses defense response (Gangappa et al., 2017). Therefore, it is possible that elevated temperatures may suppress excessive defense response in hybrid seedling lethality through such a mechanism.

The permanent application of auxin (**Figure 3E**) (Zhou et al., 1991) or temporary application of cytokinin (**Figure 3F**) (Inoue et al., 1994; Inoue et al., 1997; Kobori et al., 2007) suppresses or overcomes hybrid seedling lethality in *Nicotiana*, respectively; however, their suppression mechanisms remain unclear. Treatment of hybrid seedlings with an inhibitor of phenylalanine ammonia-lyase, L-2-aminooxy-3-phenylpropionic acid, suppressed hybrid lethality in *Nicotiana*, most likely *via* the inactivation of the salicylic acid pathway involved in defense response (**Figure 3C**) (Shiragaki et al., 2020a). Similarly, treatment of *Nicotiana* hybrid seedlings with inhibitors of ethylene biosynthesis, amino-oxyacetic acid, and amino-ethoxy vinyl glycine suppressed hybrid seedling lethality *via* the inactivation of the ethylene pathway involved in defense response (Yamada and Marubashi, 2003). Considering the tight involvement of defense response in hybrid seedling lethality, we predict that auxin and cytokinin activate the growth pathway (Vega et al., 2019), and defense responses in hybrid seedling lethality are suppressed by the crosstalk between growth and defense responses (**Figure 3**). In hybridizations between *Phaseolus vulgaris* cultivars, hybrid seedling lethality involving defense responses can be suppressed by application of cytokinin using hydroponic culture as well as by grafting of hybrid seedlings onto the parents as rootstock (Hannah et al., 2007). This may be effective because cytokinins transported from roots of the rootstocks to shoots influence growth of hybrid seedlings (Shii et al., 1981; Reiber and Neuman, 1999b). Additionally, Inoue et al. (1997) suggested other possibilities: cytokinin induces mutations in causal genes for hybrid seedling lethality or enables the screening of variant cells that carry a spontaneous mutation in the causal genes.

Irradiation with γ-rays and ion beams has been used to overcome hybrid seedling lethality (**Figure 3A**). Viable hybrid seedlings were obtained using irradiated pollen or egg cells of parents in *Nicotiana* (Shintaku et al., 1988; Shintaku et al., 1989; Kitamura et al., 2003); by irradiation of hybrid seeds in *Triticum* (Sharma, 1969) and by irradiation of hybrid shoots from the hybridization between Japanese pear and apple (Gonai et al., 2006). The tissue culture method is also effective to overcome hybrid seedling lethality (**Figure 3B**). Viable hybrid seedlings were obtained *via* calli after culture of germinated seeds, cotyledons, or leaves in *Nicotiana* (Ternovskii et al., 1972; Lloyd, 1975; Ternovskii et al., 1976; Iwai et al., 1985; Deverna et al., 1987). In *Triticum*, hybrid seedling lethality was overcome through regeneration from calli obtained by culture of immature hybrid embryos (Chen et al., 1989). Although mechanisms of overcoming hybrid seedling lethality by irradiation and tissue culture methods are not well understood, these methods may give rise to mutations or deletions of causal genes for hybrid seedling lethality or important genes in defense response during hybrid seedling lethality (**Figure 3**).

In some hybridization combinations, viable hybrids have been obtained at very low frequency without overcome and circumvent methods (Burk et al., 1979; Tezuka and Marubashi, 2006a; Tezuka et al., 2010; He et al., 2019). In some cases, this can be attributed to the deletion of the entire chromosome or certain regions of the chromosome where causal genes for hybrid lethality are located (Tezuka et al., 2010; Tezuka et al., 2012; Hancock et al., 2015). In hybridization between *N. suaveolens* and *N. tabacum*, deletion of the chromosomal region containing a hybrid seedling lethality gene could be caused by reciprocal translocations between homoeologous chromosomes of *N. tabacum* in viable hybrid seedlings obtained spontaneously very rarely as well as those obtained by tissue-culture (Nakata et al., 2021). Nevertheless, other mechanisms may also be involved in the spontaneous generation of very rare viable hybrid seedlings.

## 4 Comparison of hybrid lethality in seeds and seedlings

It would be interesting to see whether hybrid lethality in seeds and seedlings can be induced by similar mechanisms. Although much remains unknown about the genetic basis of hybrid lethality, one case shows obvious link between hybrid seed lethality and hybrid seedling lethality, i.e., hybrid lethality associated with duplicate LD1.1 and LD1.5 loci, leading to lack of histidine, as summarized above (Bikard et al., 2009). This indicates that both types of hybrid lethality are induced when the causal genes are involved in both seed and seedling stages.

However, it seems that there are no similarities in hybrid lethality between seeds and seedlings for other mechanisms. Current studies have demonstrated that in many cases, autoimmune responses are involved in hybrid seedling lethality. Nevertheless, such a mechanism has not yet been reported in hybrid seed lethality, although some cases may involve epistatic interaction as explained by BDM model. This might be attributed to the behavior of the autoimmune responses, which usually function in seedlings. The situation is similar for EBN-based hybrid seed lethality; there have been no reports stating that EBN is involved in hybrid seedling lethality. In hybridizations between *N. suaveolens* and *N. tabacum*, the use of *N. suaveolens* accessions with different EBNs did not change the severity of hybrid seedling lethality (He et al., 2019). Parental EBNs directly and indirectly affect endosperm and embryo development, respectively, in hybrid seeds but do not affect hybrid seedling development.

## 5 Possible methods to overcome or circumvent hybrid seed lethality and hybrid seedling lethality

Although several methods have been developed to overcome or circumvent EBN-based hybrid lethality caused by endosperm defects, fewer effective methods have been reported for overcoming lethality caused by defects in embryo itself. To overcome this situation, it may be possible to apply methods developed for hybrid seedling lethality (**Figure 2**). Considering that hybrid seedling lethality can be overcome by regeneration from calli formed on explants, it may be possible to overcome hybrid seed lethality through regeneration from calli induced by culture of immature hybrid embryos. In fact, hybrid seed lethality due to epistatic interaction of *Eml-A1* and *Eml-R1* can be overcome by such a method (Tikhenko et al., 2017b); however, whether this method is effective for other hybridizations remains to be investigated. Additionally, irradiation which causes DNA damage led to mutations may be effective for hybrid seed lethality as in the case of hybrid seedling lethality (**Figure 2A**). These two methods, which possibly induce new mutations, may also be effective to overcome EBN-based hybrid seed lethality because several reports have indicated that modification of the MEG or PEG expression can partially or completely rescue hybrid seeds from lethality as mentioned above. Fleming and Burrows (2023) discussed the relationships between epigenetics and mutagenesis and indicated that epigenetic modifications require DNA repair pathways for erasure, and oxidative DNA damage can alter and affect gene expression.

Several methods have been developed to overcome or circumvent hybrid seedling lethality related to autoimmune response, some of which can be effective possibly by crosstalk between defense and growth responses (**Figure 3**). Meanwhile, fewer effective methods have been reported for overcoming lethality caused by mechanisms other than autoimmune response. Hybrid seedling lethality can occur when important genes for life, such as metabolism-related genes, are redundantly copied, leading to generation of loss-of-function alleles at each locus, thereby resulting in hybrid seedlings with the set of loss-of-function alleles. This lethality can be avoided or overcome if additional substances are added to compensate for the loss of function (Bikard et al., 2009). Moreover, if it is difficult to compensate for the loss of function, the alleles at the causal loci can be changed to functional alleles using mutagens, genome editing, or genetic modification. Alternatively, cross breeding can be performed by selecting breeding lines to be free of the loss-of-function alleles.

## 6 Active application of hybrid lethality to prevent gene flow

In nature, gene flow occurs among cultivars and/or wild and weedy relatives. If hybridization occurs between cultivars or between cultivar and its relatives, genetic purity and characteristic traits of the cultivar is diminished. Furthermore, gene flow from non-transgenic herbicide resistant sorghum (*Sorghum bicolor*) and sunflower (*Helianthus annuus*) to weedy relatives is a concrete problem, although two types of strategy, physical containment (such as mesh on greenhouses and isolation distance in the field) and biological containment (such as chloroplast transformation and male sterility), have been proposed to prevent gene or transgene flow (Bozic et al., 2015; Gressel, 2015). The use of hybrid seedling lethality genes has been proposed to prevent genetic contamination (Sato and Inarura, 1989; Yonezawa et al., 1990). To achieve this, the cultivar possessing an epistatic gene which causes hybrid seedling lethality with its counterpart is developed. When the cultivar is pollinated by pollen from other cultivars or its relatives which possess the counterpart gene, hybrid seedlings show hybrid lethality and the inflow of alien genes to the cultivar can be suppressed. In this system, a set of epistatic genes causing F_1_ hybrid seedling lethality is available, but those causing hybrid breakdown are not useful. Such a system would also be effective to suppress outflow of foreign genes in transgenic plants to cultivars and wild and weedy relatives in the field.

For practical application of this system, sufficient severity of hybrid seedling lethality is required to prevent progeny from surviving (Sato and Inarura, 1989; Yonezawa et al., 1990). Additionally, high stability of hybrid seedling lethality would be required because viable hybrids might emerge at very low frequency as mentioned above in interspecies crosses in *Nicotiana*. Therefore, use of additional set of epistatic genes causing hybrid seedling lethality might be effective. In *Nicotiana*, very rare survival hybrids seem unlikely to emerge when two sets of epistatic genes are possibly involved in hybrid seedling lethality (Tezuka and Marubashi, 2012). Additionally, use of other isolation mechanisms may also be effective, although there may also be loopholes in these mechanisms. For example, germinable seeds may be rarely produced even though the hybridization combination almost always produces hybrid seeds showing EBN-based hybrid seed lethality (He et al., 2019). However, combination of hybrid seed lethality and hybrid seedling lethality is expected to suppress gene flow more robustly than each lethality alone.

## 7 Conclusions and perspectives

Hybrid lethality has been considered to occur in embryos and seedlings after hybridizations between different lineages. However, similarities and differences between hybrid seed lethality and hybrid seedling lethality remain poorly understood. Recent molecular evidences indicated that evolution of genes derived from a common ancestor can lead to deleterious epistatic interaction leading to both hybrid seed lethality and hybrid seedling lethality. Additionally, hybrid seed lethality involves a unique mechanism related to endosperm, whereas hybrid seedling lethality involves a unique mechanism related to autoimmune response. For plant breeding, hybrid lethality is an obstacle which needs to be overcome or circumvented, as well as a valuable resource to prevent gene flow. In this context, it is necessary to identify other genes involved in hybrid lethality and investigate whether other mechanisms are involved in hybrid lethality.

## Author contributions

TT proposed the concept. All authors conceived the manuscript. HH and TT wrote the manuscript. KS wrote part of the manuscript regarding methods to overcome hybrid seedling lethality. HH and KS prepared the figures. All authors reviewed and edited the manuscript. All authors have read and agreed to the published version of the manuscript.
